# Environmental hydro-refugia demonstrated by vegetation vigour in the Okavango Delta, Botswana

**DOI:** 10.1038/srep35951

**Published:** 2016-10-24

**Authors:** S. C. Reynolds, C. G. Marston, H. Hassani, G. C. P. King, M. R. Bennett

**Affiliations:** 1 Institute for Studies in Landscape and Human Evolution, Faculty of Science and Technology, Bournemouth University, Fern Barrow, Poole, BH12 5BB, UK; 2 Nottingham University, University Park, Nottingham, NG7 2RD, UK; 3 Edge Hill University, Ormskirk Campus, Ormskirk, L39 4QP, UK; 4 Institute for International Energy Studies, Tehran 1967743711, Iran; 5 Laboratoire de Tectonique, Institut de Physique du Globe de Paris, Paris Cedex 05, France

## Abstract

Climate shifts at decadal scales can have environmental consequences, and therefore, identifying areas that act as environmental refugia is valuable in understanding future climate variability. Here we illustrate how, given appropriate geohydrology, a rift basin and its catchment can buffer vegetation response to climate signals on decadal time-scales, therefore exerting strong local environmental control. We use time-series data derived from Normalised Difference Vegetation Index (NDVI) residuals that record vegetation vigour, extracted from a decadal span of MODIS images, to demonstrate hydrogeological buffering. While this has been described previously it has never been demonstrated via remote sensing and results in relative stability in vegetation vigour inside the delta, compared to that outside. As such the Delta acts as a regional hydro-refugium. This provides insight, not only to the potential impact of future climate in the region, but also demonstrates why similar basins are attractive to fauna, including our ancestors, in regions like eastern Africa. Although vertebrate evolution operates on time scales longer than decades, the sensitivity of rift wetlands to climate change has been stressed by some authors, and this work demonstrates another example of the unique properties that such basins can afford, given the right hydrological conditions.

Rift basins and associated wetlands may offer attractive habitats and their role in human evolution has been stressed by several authors^1^,^2^,^3^,^4^. The Okavango Delta is the largest wetland in southern Africa and renowned for its high floral and faunal biodiversity^5^. It covers an area of over 40,000 square km and consists of a smooth, (relief ≤2 m; slope 1:3400) conically-shaped, alluvial fan located on the Cubango-Okavango River which feeds a network of distributary channels and flanking swamps that form the Delta itself^6^,^7^. While more accurately an alluvial megafan (or fan delta) rather than a delta we preserve the current terminology to avoid confusion. Located in the Kalahari Basin, the region has been subject to sedimentation throughout the Cenozoic, and was once at the centre of palaeo-lake Makgadikgadi^8^,^9^,^10^,^11^,^12^, prior to its drainage as a result of continued tectonic activity associated with the westward propagation of the East African Rift and river capture by the Zambezi River^10^,^13^,^14^. The Delta is now located in an asymmetric graben, demarcated by a series of sub-parallel faults, including the Thamalakane Fault and is in-filled with at least 300 m of sediment deposited sometime in the last 1 Ma^10^,^15^,^16^,^17^. Podgorski and colleagues^12^ report a combination of electromagnetic, borehole and seismic data and suggest that this sediment fill consists of palaeo-megafans in the Okavango Basin overlain, by palaeo-lake Makgadikgadi deposits and the current delta/megafan. Recent renewed movements, post the palaeo-lake, along the Thamalakane and Kunyere faults now separate the Okavango and Makgadikgadi basins^10^. A series of topographic cross-sections illustrate the geomorphological features of the Delta and wider basin.

The Okavango catchment is dominated by aeolian sands and outcrops of weathered bedrock (Fig. 1). The annual sediment discharge within the Cubango-Okavango River consists mainly of bed and solute loads (bed: 170,000 tonnes; solute: 360,000 tonnes) while suspended sediment forms only a minor component (8 mg/l or 39,000 tonnes)^18^,^19^. As a consequence of the high sediment porosity (very well-sorted with rounded grains) over 80 to 90%^20^ of seasonal flood water infiltrates into the Delta from the Cubango-Okavango River with peak flows in the Panhandle in April and early May (Fig. 1). Much of this shallow water is then transpired by Delta vegetation as this slow moving flood wave takes over four months to reach the south eastern extremity of the Delta (i.e., in August/September). Total discharge is the order of 10 cubic km of water with an additional 6 cubic km of rainfall falling between October and May each year (300 to 1000 mm per annum over the last 70 years^7^, sustaining 2412 square km of permanent wetland and a further 5927 square km of seasonal wetland^20^. The proportion of water that escapes the Delta is uncertain, some estimates place this as little as 2%^20^ although it could be higher, however in practice the majority infiltrates into the alluvial fan, forming a near-surface groundwater body (circa. 8 m) sitting above and surrounded by older, more saline, groundwater in deeper hydrological units^12^. Water loss is via evapotranspiration, particularly associated with trees (circa. 25% loss, from just 7% of the surface area) that fringe inter-channel islands constructed of salt^12^. Groundwater flow is directed towards the islands and salt is concentrated at the centres in sufficient concentrations to inhibit vegetation growth^20^. The pattern of annual rainfall derived from Tropical Rainfall Measuring Mission (TRMM) satellite derived data shows a northwest to southeast trend decreasing from 700 mm to 590 mm in the southeast (Supplementary Information, Fig. S1). The hydrological system of the delta consists of surface water, saturated soils and a near-surface water table; we refer to this as an integrated surface-ground water system. Our aim is to show how these distinctive hydrological properties of the Okavango reduce the amplitude of seasonal and decadal variations in vegetation vigour inside the Delta extent, and consequently, enhance its capacity to buffer climate, on at least decadal timescales.

## Methods

Data was collected for the Okavango Delta using time-stacked satellite imagery from the Moderate Resolution Imaging Spectroradiometer (MODIS) sensor. MODIS Normalized Difference Vegetation Index (NDVI) 16-day composites at 250 m resolution (MOD13Q1) imagery was acquired from the United States Geological Survey, providing complete temporal coverage from 2001 to 2013 (N = 299; Supplementary Information Table S1). The MODIS Re-projection Tool was used to subset the MODIS imagery to the study area extent and to re-project all data into UTM WGS84 zone 34 South projection. The images were composited in time-order to extract a phenological profile of NDVI growth and senescence for every pixel within the image extent. To smooth the phenological profile for each pixel a harmonic analysis, using an inverse Fourier Transform, was performed^21^. Taking just a single location as an example, this works by fitting a series of sine waves to the dataset to characterise vegetation cycles in the data over time–these could be the expected seasonal cycle, longer term multi-annual cycles or even sub-annual cycles. The series of sine curves will characterise the main phenological profile of the site, while the remaining residuals will correspond to unusual variability in vegetation levels from, for example, more extensive flooding and vegetation growth as well as slightly more vigorous vegetation flushes in wetter years, the opposite in drier years and the effects of fire, for example. In this way, a map of residuals was created (Fig. 2). A series of randomly located sample points across the study area was then generated, with 200 points located within the wetland extent and a further 200 outside the wetland extent. The wetland extent was determined using four 30 m resolution Landsat ETM + images from the wet season (March-April) (Supplementary Information, Table S2, Fig. S2). This showed the most pronounced vegetation contrast signal across the wetland area allowing its extent to be mapped. By doing so, delineated the random points lying within, and without, the wetland areas. Given the 250 m pixel size of MODIS, points that fell close (±250 m) to the wetland extent boundary were disregarded to avoid potential problematic mixed-pixel effects (Supplementary Information, Table S3). The unsmoothed time-series NDVI and TRMM data values were then extracted for each sample point.

To establish corresponding spatio-temporal patterns in rainfall across the study area, TRMM data (product 3B43) was used. This is a monthly product with a spatial resolution of 0.25° × 0.25°, and is a best estimate of precipitation rate based on 3-hourly merged high-quality satellite estimates and monthly accumulated Global Precipitation Climatology Centre (GPCC) rain gauge analysis. Complete temporal coverage of this data product from 2001 to 2013 was acquired (Supplementary Information, Figures S1).

Data extracted from the 400 random points were subject to a Singular Spectrum Analysis (SSA). The aim of SSA is to make a decomposition of the original series into the sum of a small number of independent and interpretable components, such as a slowly varying trend, oscillatory components and a structure-less noise^22^,^23^. There are two stages: first, we decompose the series and then reconstruct it, but minus the noise. This reconstructed series can then be used if required, to forecast new data points. This analysis was conducted in R by one of the authors and follows published methods^24^,^25^.

### Stage 1: Decomposition

*1st step: Embedding:* Embedding can be regarded as a mapping step that transfers a one-dimensional time series *Y*_*T*_ = (*y*_1_, …., *y*_*T*_) into the multi-dimensional series X_1_, …, X_K_ with vectors *X* = (*y*_1_, …, *y*_*i*+*L*−l*i*_)′∈*R*^*L*^, where *K* = *T*−*L* + 1. The single SSA choice (parameter) of the embedding step is the *window length L*, an integer such that 2 ≤ *L* ≤ *T*−1. The result of this step is the trajectory matrix 
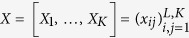
. Note that the trajectory matrix X is a Hankel matrix, which means that all the elements along the diagonal *i* + *j* = *const* are equal.

*2nd step: Singular Value Decomposition (SVD):* The second step, the SVD step, makes the singular value decomposition of the trajectory matrix and represents it as a sum of rank-one bi-orthogonal elementary matrices. Denote by *λ*_1_, …. *λ*_*L*_ the eigenvalues of XX’ in decreasing order of magnitude (*λ*_1_≥ … *λ*_*L*_≥0), by *U*_1_, …. *U*_*L*_ the orthonormal system and set


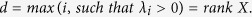


If we denote 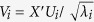, then the SVD of the trajectory matrix can be written as:


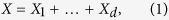


where 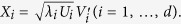 The collection 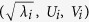 is called the *i*-th eigentriple of the matrix 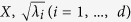 are the singular values of the matrix *X* and the set {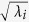} is called the spectrum of the matrix *X*.

### Stage 2: Reconstruction

*1st step: Grouping*: The grouping step corresponds to splitting the elementary matrices *X*_*i*_ into several groups and summing the matrices within each group. Let *I* = {*i*_1_, …. *i*_*p*_} be a group of indices *i*_1_, …. *i*_*p*_. Then the matrix *X*_*i*_ corresponding to the group *I* is defined as *X*_*I*_ = *X*_*i*1_ + … + *X*_*ip*_. The split of the set of indices *J* = 1, …. *d* into the disjoint subsets *I*_1_, …. *I*_*m*_ corresponds to the representation


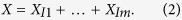


*2nd step: Diagonal Averaging:* Diagonal averaging transfers each matrix *X*_*I*_ into a time series, which is an additive component of the initial series *Y*_*T*_. If *z*_*ij*_ stands for an element of matrix *Z*, then the *k*-th term of the resulting series is obtained by averaging *z*_*ij*_ over all *i*, *j* such that *i* + *j* = *k* + 2. This procedure is called *diagonal averaging*, or Hankelization of the matrix *Z*.

**Figure 1:**
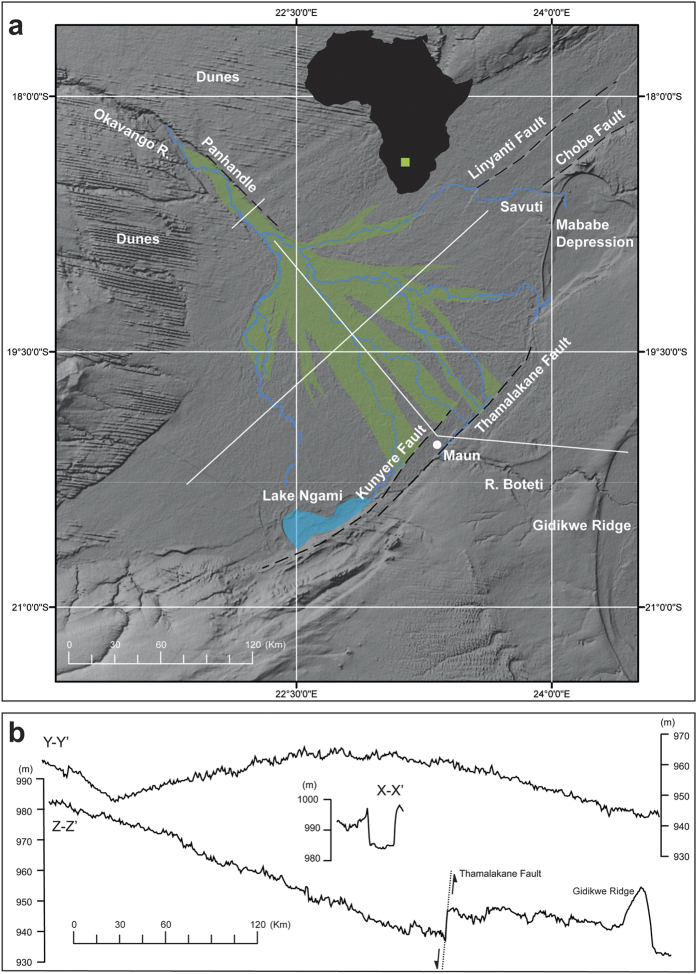
The Okavango Delta region. (**a**) Hill shaded digital elevation model for the Okavango Delta showing the delta, key locations, channels and faults. From ALOS Global Digital Surface Model with a 30 m resolution (http://www.eorc.jaxa.jp/ALOS/en/aw3d30/index.htm) processed in ESRI ArcMAP 10.1 (http://desktop.arcgis.com/en/arcmap/). © JAXA. (**b**) Relief ross-sections derived from ALOS Global Digital Surface Model with a 30 m resolution (http://www.eorc.jaxa.jp/ALOS/en/aw3d30/) processed in ESRI ArcMAP 10.1 (http://desktop.arcgis.com/en/arcmap/).

**Figure 2:**
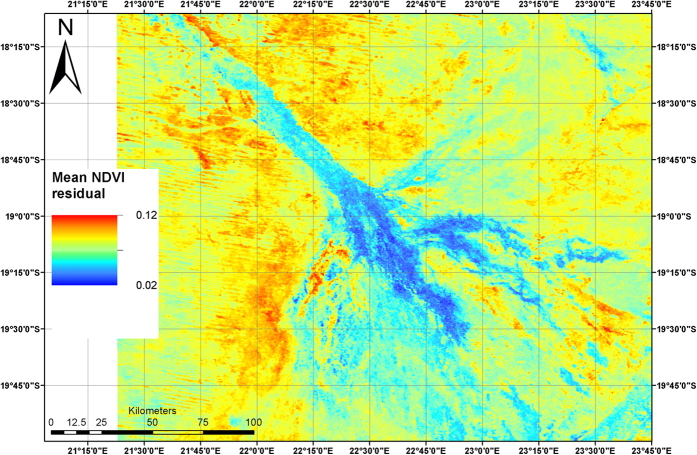
NDVI residuals for a decadal span (2001–2013) derived from MODIS imagery for the Okavango Delta. Note the low residuals (blue) inside the alluvial system compared to those outside (red/yellow). The image was created using MODIS data in IDRISI Selva version 17.01 (https://clarklabs.org/) and R version 2.13.1 (https://www.r-project.org/). The smaller residuals tend to occur in areas dominated by *Cyperus papyrus* and *Phragmites spp*., and aquatic macrophytes characteristic of perennially inundated areas. The lighter shades of blue correspond to regularly seasonally inundated floodplains dominated by a mixture of grasses and sedges. In these latter, the water is regularly drawn down beneath the soil surface. The highest of residuals correspond to dryland areas. Where islands are small, the pixel size of MODIS can result in the loss of these essentially terrestrial features, producing a mixed signal despite appearing to lie “inside” the wetland.

**Figure 3:**
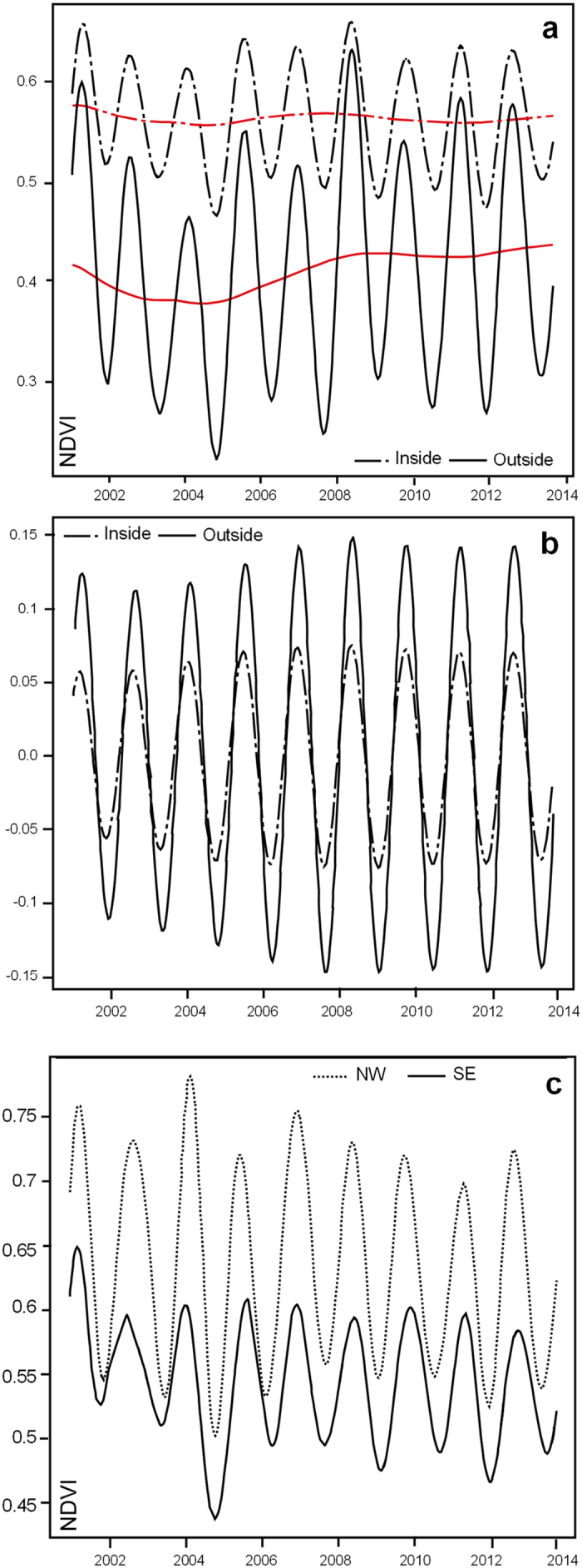
Compilations of time-series data for randomly sampled points inside and outside for the Okavango Delta. (**a**) Reconstructed time series data showing the decadal trend. (**b**) Reconstructed time series data with the decadal trend removed. (**c**) Time series for two points randomly selected at either end of a NW to SE transect starting at the panhandle and bisecting the Okavango Delta. The SSA analysis was performed in R version 2.13.1 (https://www.r-project.org/).

The window length *L* is the only SSA choice in the decomposition stage. Given that the time series is likely to have a periodic component with an integer period (i.e. seasonal component) better ‘separability’ is achieved by using a window length proportional to that period (there are 45.6 satellite over passes per year). Consequently, we took *L* = 46 which using the SVD gives a trajectory matrix 46 × 46) and 46 eigentriples ordered by their contribution (share) in the decomposition (Supplementary Information, Fig. S3). Figure 3A and B depicts the plot of the logarithms of the 46 singular values for the Okavango Delta. Only one of the evident pairs has almost equal leading singular values and this corresponds to one (almost) harmonic component in both the inside and outside series for the Okavango Delta (eigentriple pairs 2–3) which corresponds to a period of 23 months (Supplementary Information, Fig. S5). The decadal trend was obtained from the first eigentriple (Supplementary Information, Fig. S6). Extraction of residuals (i.e. minus trend and oscillation) equate to the noise allowing final reconstruction of the data series (Supplementary Information, Figs S7 and S8; Fig. 1B). Summary data for the two time series is provided in Table 1A,B. The main concept in studying SSA properties is ‘separability’, which characterises how well different components can be separated from each other^23^,^24^. The weighted correlation (or w-correlation) is a natural measure of dependence between two series 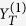
and 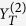:


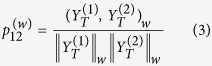


where 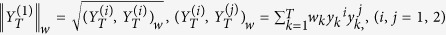



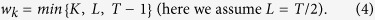


If two reconstructed components have zero *w*-correlation it means that these two components are separable. Large values of *w*-correlations between reconstructed components indicate that the components should possibly be gathered into one group and correspond to the same component in SSA decomposition. Table 1C contains the weighted correlation between signal and noise for the Okavango Delta time series decomposition and reconstructions with SSA. It is clear that we have obtained a sound ‘separability’ between signal and noise as the *w*-correlations are close to zero in all instances.

## Results

**Figure 4:**
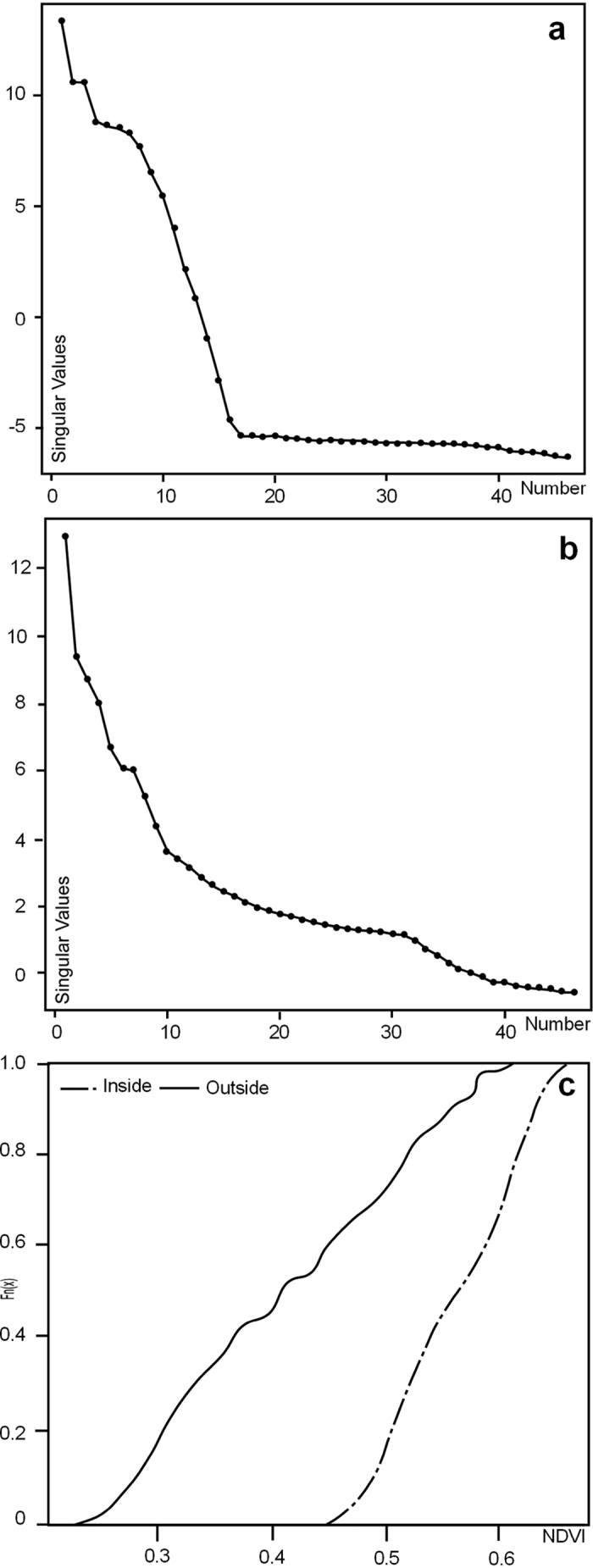
(**a**) Logarithms of the 46 eigenvalues for the outside data series. (**b**) Logarithms of the 46 eigenvalues for the inside data series. (**c**) Empirical cumulative distribution functions (CDF’s) for the two time series showing their distinctive character. The SSA analysis was performed in R version 2.13.1 (https://www.r-project.org/).

The time stacked NDVI residuals reveal contrasts in both the amplitude of seasonal, and inter-year, variation in the vegetation vigour (enhanced growth and extent) and also between areas inside, and outside, of the Okavango Delta (Fig. 4A). Both show similar patterns in terms of the direction of change associated with the seasonal rainfall regime, but vary in absolute value, seasonal amplitude and degree of variance (Fig. 4A; Table 1A,B). Mean values are similar for the two time-series (inside and outside) although the coefficient of variance shows the values are more stable outside the delta (Table 1A). It is clear from Fig. 3 that both series follow a similar pattern in terms of the direction of change, although it is also evident that both series have different amplitudes with the outer series showing greater variation. Towards 2013 both series show a declining trend. In Fig. 3B the decadal trend has been removed.

The empirical cumulative distribution functions (CDFs) for the reconstructed pattern of inside and outside series is visibly different (Fig. 3C) and using a two-sample Kolmogorov-Smirnov (KS) test can be shown to be significantly different statistically (KS Test Statistic = 0.6716 P < 0.01). In summary, the data shows that while there is considerable inter-point variance in NDVI values within the Okavango Delta, consistent with the complex channel and island vegetation patterns present^26^,^27^, seasonal and decadal variation are suppressed. The properties of the Okavango Delta appear therefore to repress (buffer) the seasonal and decadal climate signal.

### Interpretation

The absolute contrast in NDVI values between those inside the Delta with those outside, reflect differences in the composition and type of the vegetation^27^. Within the delta the high NDVI values reflect the wetland macrophytes which are a function of the surface water and subsequently soil moisture during the flood wave^28^. Groundwater plays a role in maintaining both surface water and elevated soil moistures as the flood wave wanes. The riparian woodland fringe (*circa*. 30 m) on some delta islands while significant in terms of evapotranspiration (circa. 25%) has a relatively small extent and is unlikely to be picked out in the NDVI pixels.

The contrast in seasonal amplitude between the inside and outside points is a function of two factors. Firstly, the arrival of the flood wave within the delta lags the local climatically-induced wet season. This occurs because under present conditions, 95% of the flow of the Okavango River that feeds the Delta comes from only a quarter of the total catchment (i.e. Angolan Highlands) approximately 1000 km to the north^27^. Peak rains in the Angolan Highlands occur in December through to February each year, but only reach the panhandle at Mohembo around the middle of April or in early May (Fig. 1). The catchment also provides potential for hydrological buffering. Water storage occurs in the arenosols and weathered saprolytes of the Angolan catchment, where slopes are gentle, and soils deep, sandy, and well covered by woodland^29^. Here much of the incident rainfall infiltrates the soil to recharge phreatic aquifers, from which water moves laterally to emerge via wetlands drained by small streams. The rate of groundwater discharge from these shallow local aquifers is sufficiently slow that they provide a degree of inter-year buffering of base flow.

Secondly, the slow passage of the flood wave within the Delta itself which is a function of the rifted (tectonic) basin, and its associated high porosity sediment fill, dampens the seasonal signal further. As the flood wave wanes recharge from shallow groundwater recharge maintains the surface waters and elevated soil moisture. Cyclical variations in channel vegetation, due perhaps to nutrient load may complicate the signal locally^30^. There is a small systematic time lag in peak NDVI along the long axis of the delta which reflects the four month progression of the annual flood wave^19^. Points lying approximately along a northwest to southeast axis provide data to examine this. As illustrated in Fig. 3C seasonal amplitude in NDVI declines to the south east reflecting the presence of increased standing water and associated changes in vegetation structure.

Multi-decadal oscillations (30 + years) in the hydrology of the Okavango River have been documented by Wolski and colleagues^31^. Quasi-periodic fluctuations with 16–18 and 60–80 years as well as shorter 3–8 year cycle lengths have been reported^7^,^32^. These have been tentatively correlated to variation primarily in rainfall and potential evapotranspiration and North Atlantic Oscillations (NAO) and Pacific Decadal Oscillations (PDO)^33^,^34^,^35^. Multi-year oscillations are visible in the NDVI data reported here; however we note that these oscillations are subdued, in comparison to those outside the delta. We suggest that the most parsimonious explanation for this is some form of groundwater buffering, in the broadest sense of this term. It therefore results from the distinctive hydrogeological setting of the rift basin (and also in this case the catchment) and the associated surface water and near–surface aquifers. While recognition of this buffering capacity is not necessarily new^36^,^37^, demonstrating its extent and significance via NDVI is. It is worth noting that the buffering capacity has historically made modelling of the system difficult. Early hydrological models of the Delta attributed the inability to produce consistent simulations of outflows based on inflow to the redistribution of flow in the complex channel systems^36^,^37^. More recent models have explicitly incorporated groundwater storage^38^ and the parameterisation of this has steadily improved^39^,^40^.

### Implications

The data presented here suggest that the distinctive hydrogeological properties of the Okavango Basin and catchment buffer seasonal and decadal climate variations. Near-surface groundwater storage releases moisture to vegetation, both within years and between years, and accounts for the subdued seasonal variations in NDVI observed. While these properties are, in part unique to the Okavango system in that the tectonic basin contains porous aeolian sands and the proportion of transported fines (silt + clay) in the Okavango River from weathered bedrock is small, we suggest that other tectonic basins may show similar capacity for groundwater buffering given appropriate sedimentary fills. Active tectonics creates the ‘accommodation space’ for sedimentation, in this case, at least two, mega-fans^10^. Terrestrial rift basins are commonly in-filled by coarse alluvial fans that have the potential to form aquifer-rich units. The asymmetry of the tectonic Okavango Basin, located at the western edge of the propagating East African Rift, is similar to other sections of the rift, both in the geological past and present^41^. Asymmetrical grabens are conducive to the development of large fan systems, like that in the Okavango Basin, and consequently similar conditions may have prevailed in the past elsewhere along the rift.

During the Quaternary, the Okavango Basin and the surrounding areas was not only a significant wetland but also characterised by periods in which mega-lakes formed^8^. Hydrological re-organisation was caused by changes in rainfall volumes in the Angolan Highlands and the lakes themselves may have been of sufficient size to influence local climate^42^. They would have supported a rich flora around their margins attractive to a diverse fauna. Podgorski and colleagues^12^ found evidence for at least one mega-fan potentially pre-dating these lake deposits and, at low lake stands, fans of various sizes are likely to have been present, and such deposits may interdigitate with the lake deposits. These fans, if similar to that of the Okavango today, may have been very important as refugia during these drier intervals as ‘wetlands in drylands’^43^.

The degree to which the Okavango Basin’s hydrological/hydrogeological properties are unique, or at least distinctive, is open to debate. The distinct suite of properties include: extended separation of catchment source from the sink; the deeply weathered catchment which buffers inter-annual base flow; the aridity of the surrounding areas; the porous basin-fill; when coupled with the active tectonics creating the ‘accommodation space’ for the lake and fan sediments and perhaps driving the reorganisation of the regional drainage system leading to lake drainage. They have made the basin an area of hydrological importance in the past and give the Delta today its distinctive vegetation, which supports a rich fauna of international importance^44^ and which shows evidence of evolutionary vicariance in at least one antelope taxon^45^. We suggest that one of the contributing factors to this rich vegetation is the capacity of the hydrological and hydrogeological system to buffer seasonal and decadal variations in vegetation vigour.

The idea that a tectonic basin, or more accurately the sedimentary infill within the accommodation space created by that basin, may exert a control on the fauna and flora within that basin at least on a decadal time-scale, may have wider implications. For example, long-term effects of uplift and rifting on regional climate are well-established, especially for areas of eastern Africa^46^, however the effects documented here are more local, and operate over much shorter time scales, but are relevant to the potential of these tectonic basins to operate as climatic refugia for both flora and fauna during periods of adverse climate. Elsewhere, tectonics has been implicated in creating habitats suitable and attractive for repeated episodes of hominin occupation over longer timescales across Africa and worldwide^1^,^2^,^47^. The Olduvai Gorge (Tanzania) provides one example, amongst many, of active tectonic basins which were repeatedly occupied by hominins^48^,^49^,^50^. Topographic roughness, the availability of water and the potential for the formation of wetlands (with associated high biodiversity and consequently a range of food resources), would have been key advantages of such locations to hominins. To this we can now add the potential of such areas to provide short-term climate refuge against environmental extremes, provided suitable basin fills were available. A diversity of topographic basins, some tectonically active and others passive, offer a range of different topographically-controlled responses to similar climate signals which, if sustained over prolonged periods, may have led to environmental contrasts that could have favoured evolutionary processes such as vicariance and speciation. The Okavango Delta also clearly shows the links between wetlands, biodiversity and ecosystems services for hominin and human survival in the present, however, it is likewise important because this region has experienced relatively little anthropogenic transformation and so may help illuminate the functioning of past landscape with which our hominin ancestors were associated.

## Additional Information

**How to cite this article**: Reynolds, S. C. *et al*. Environmental hydro-refugia demonstrated by vegetation vigour in the Okavango Delta, Botswana. *Sci. Rep*. **6**, 35951; doi: 10.1038/srep35951 (2016).

## Supplementary Material

Supplementary Information

## Figures and Tables

**Table 1:** Data for the Okavango Delta.

Part A		OKD-Inside	OKD-Outside
Mean	Mean	5613.73	4159.65
	Med.	5650.53	4164.84
	S.D.	593.62	437.10
	CV	10.57	10.50
	S-W	0.19^a^	0.59^a^
	ADF (tau)	−0.63^b^	−0.49
Median	Mean	5613.29	4107.82
	Med.	5649.82	4105.26
	S.D.	597.97	463.49
	CV	10.65	11.28
	S-W (p-value)	0.22^a^	0.71^a^
	ADF (tau)	−0.63^b^	−0.52^b^
S.D	Mean	651.12	1121.11
	Med.	626.33	1145.41
	S.D.	222.40	219.58
	CV	34.15	19.58
	S-W (p-value)	<0.01	<0.01
	ADF (tau)	−1.97*	−1.17*
Part B			
	Mean	2813.97	4413.02
	Median	2669.92	4490.61
	S.D.	822.91	762.83
	CV	29.24	17.28
	S-W (p-value)	<0.01	<0.01
	ADF (tau)	−1.69^c^	−1.11^c^
Part C			
	Series	OKD-Inside	OKD-Outside
	Signal	r = (1:3)	r = (1:3)
	Residual	r = (4:46)	r = (4:46)
	w-corr.	0.006	0.013

Part A: Descriptive statistics for the Okavango time series. Part B: Descriptive statistics for the amplitude in Okavango time series. Part C: w-correlations between signal and noise for Okavango time series.

Notes Part A: ^a^indicates data is normally distributed based on a Shapiro-Wilk test at p = 0.01.

^b^Indicates data is non-stationary based on the Augmented Dickey-Fuller test at p = 0.01.

Note Part B: ^c^indicates data is non-stationary based on the Augmented Dickey-Fuller test at p = 0.01.

Notes Part C: A window length, L = 46 has been used for all series and r represents the number of eigenvalues used for reconstruction of the filtered series.
