# Supplementary material for: Environmental hydro-refugia demonstrated by vegetation vigour in the Okavango Delta, Botswana
Source: Sci Rep. 2016 Oct 24;6:35951. doi: 10.1038/srep35951 (PMC5075895; doi:10.1038/srep35951)
Supplement: Supplementary Information [file srep35951-s1.doc]

**Supplementary Information for:** **Environmental hydro-refugia demonstrated by vegetation vigour in the Okavango Delta, Botswana**

**Reynolds S.C.^1^, Marston, C.G.^2,3^, Hassani, H.^1^, King G.C.P.^4^,and Bennett M.R.^1^**

*^1^Institute for Studies in Landscape and Human Evolution, Faculty of Science and Technology, Bournemouth University, Fern Barrow, Poole, BH12 5BB, UK.*

*^2^Nottingham University, University Park, Nottingham, NG7 2RD, UK.*

*^3^ Edge Hill University, Ormskirk Campus, Ormskirk, L39 4QP, UK*

*^4^Laboratoire de Tectonique, Institut de Physique du Globe de Paris, Paris Cedex 05, France.*

Additional tables and figures pertaining to the remote sensing and time series analyses.

*Table S1:* MODIS 16-day compositing period imagery acquisition dates.

| **16-day compositing period** | **Julian Day start and finish** | **Calendar Day (non-leap year)** | **Calendar Day (leap year )** |
| --- | --- | --- | --- |
| 1 | 1 – 16 | Jan 01 – Jan 16 | Jan 01 – Jan 16 |
| 2 | 17 – 32 | Jan 17 – Feb 01 | Jan 17 – Feb 01 |
| 3 | 33 – 48 | Feb 02 – Feb 17 | Feb 02 – Feb 17 |
| 4 | 49 – 64 | Feb 18 – Mar 05 | Feb 18 – Mar 04 |
| 5 | 65 – 80 | Mar 06 – Mar 21 | Mar 05 – Mar 20 |
| 6 | 81 – 96 | Mar 22 – Apr 06 | Mar 21 – Apr 05 |
| 7 | 97 – 112 | Apr 07 – Apr 22 | Apr 6 – Apr 21 |
| 8 | 113 – 128 | Apr 23 – May 08 | Apr 22 – May 07 |
| 9 | 129 – 144 | May 09 – May 24 | May 08 – May 23 |
| 10 | 145 – 160 | May 25 – Jun 09 | May 24 – Jun 08 |
| 11 | 161 – 176 | Jun 10 – Jun 25 | Jun 09 – Jun 24 |
| 12 | 177 – 192 | Jun 26 – Jul 11 | Jun 25 – Jul 10 |
| 13 | 193 – 208 | Jul 12 – Jul 27 | Jul 11 – Jul 26 |
| 14 | 209 – 224 | Jul 28 – Aug 12 | Jul 27 – Aug 11 |
| 15 | 225 – 240 | Aug 13 – Aug 28 | Aug 12 – Aug 27 |
| 16 | 241 – 256 | Aug 29 – Sep 13 | Aug 28 – Sep 12 |
| 17 | 257 – 272 | Sep 14 – Sep 29 | Sep 13 – Sep 28 |
| 18 | 273 – 288 | Sep 30 – Oct 15 | Sep 29 – Oct 14 |
| 19 | 289 – 304 | Oct 16 – Oct 31 | Oct 15 – Oct 30 |
| 20 | 305 – 320 | Nov 01 – Nov 16 | Oct 31 – Nov 15 |
| 21 | 321 – 336 | Nov 17 – Dec 02 | Nov 16 – Dec 01 |
| 22 | 337 – 352 | Dec 03 – Dec 18 | Dec 02 – Dec 17 |
| 23 | 353 – 365+ | Dec 19 – Jan 03 | Dec 18 – Jan 02 |


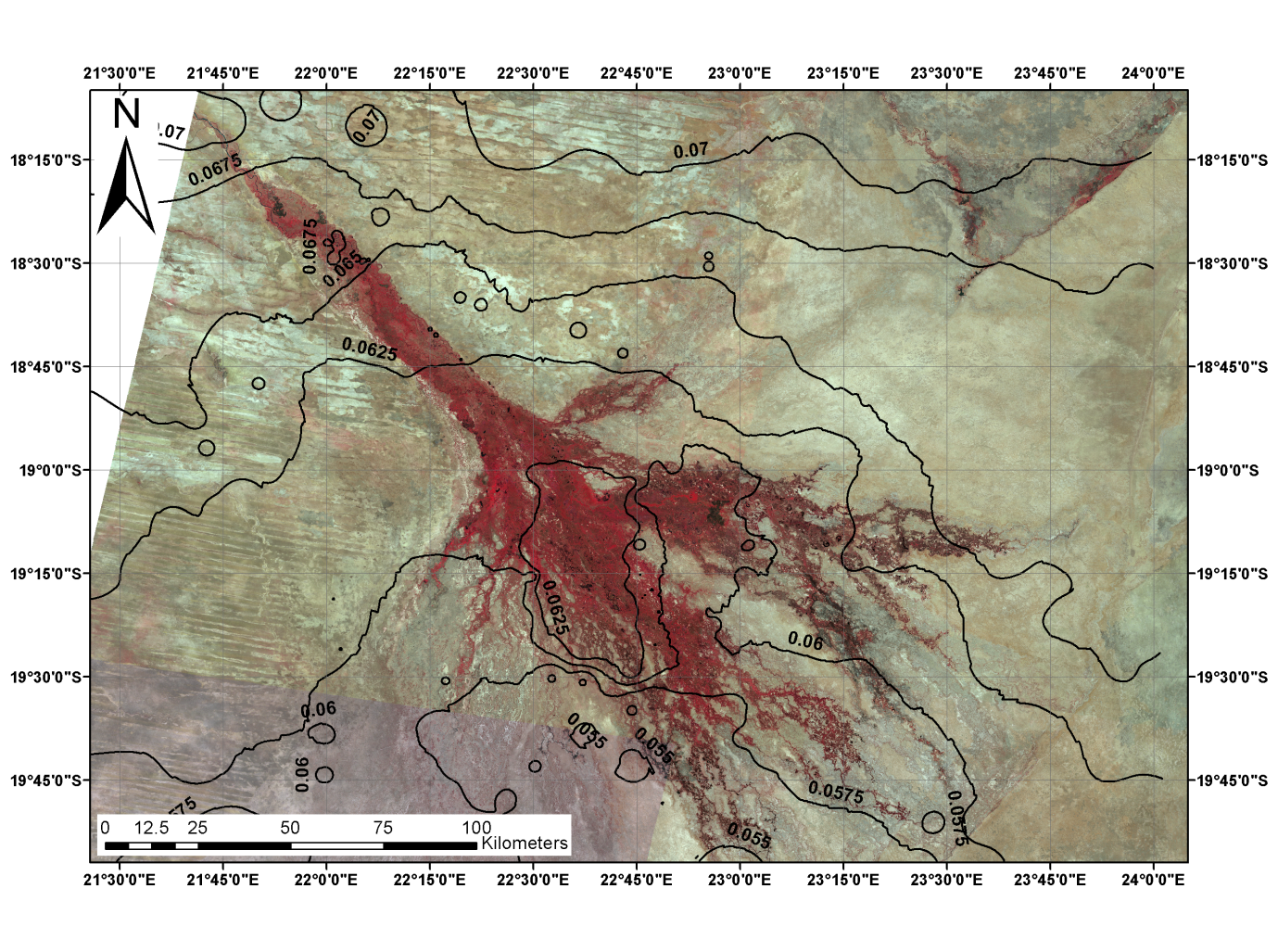
*Figure S1: Plot of isohyets (m/annum) for the Okavango Delta derived from TRMM data The image is a false-colour composite where areas of vegetation show is as red. This was created using Landsat bands 4 (near infrared), 3 (red) and 2(green). The image was created using TRMM data in IDRISI Selva version 17.01 (https://clarklabs.org/) and R version 2.13.1 (https://www.r-project.org/).*


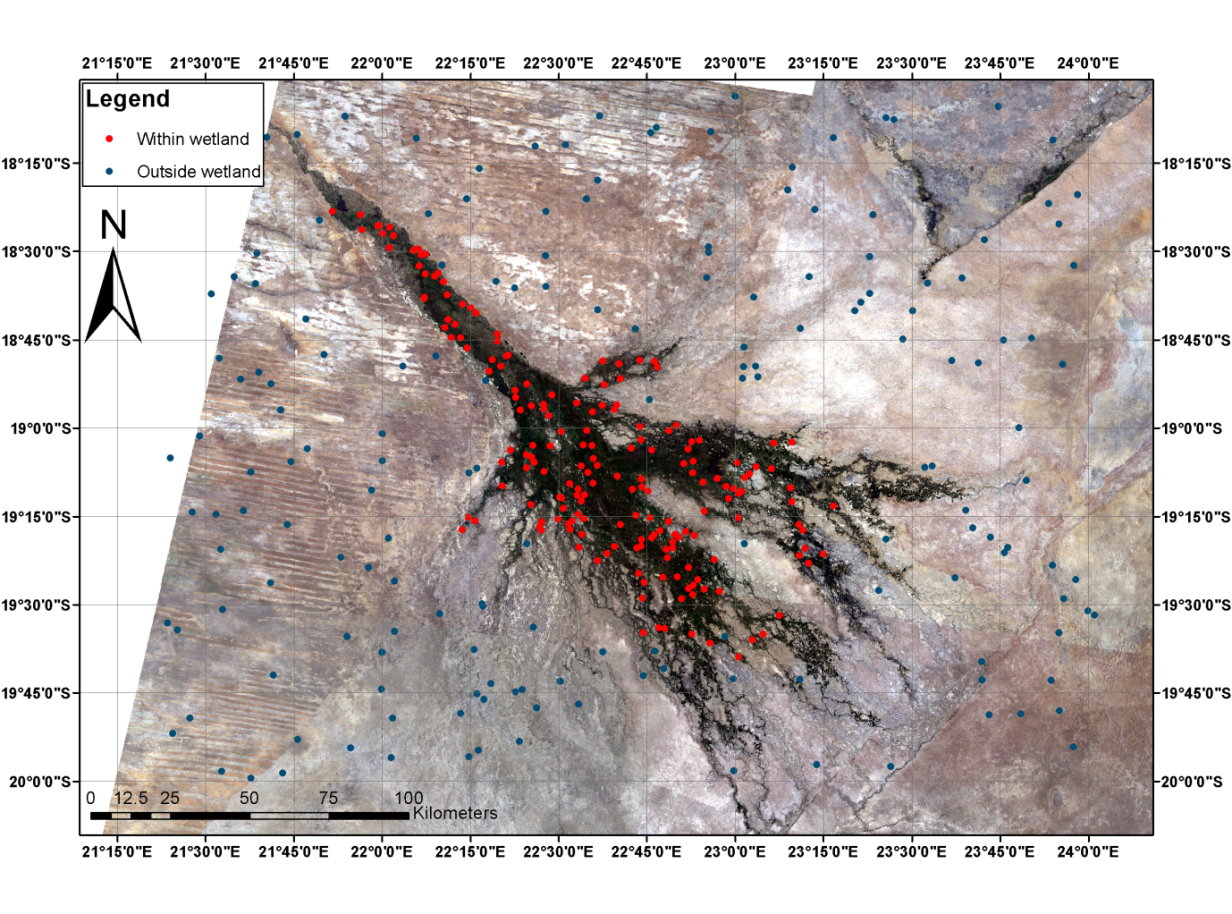


*Figure S2 Random sample points inside (red) and outside (blue) for the Okavango Delta. The image is a true-colour composite where vegetated areas within the wetland show us as dark green. This was created using bands 3 (red), 2 (green), and 1 (blue).The image was created using MODIS data in IDRISI Selva version 17.01 (https://clarklabs.org/) and R version 2.13.1 (https://www.r-project.org/).*

*Table S2* Landsat ETM+ image acquisition dates.

| **Site** | **Landsat path and row** | **Image acquisition date** |
| --- | --- | --- |
| Okavango | path 174, row 73 | 10^th^ October 1999 |
| Okavango | path 175, row 73 | 2^nd^ November 1999 |
| Okavango | path 174, row 74 | 10^th^ October 1999 |
| Okavango | path 175, row 74 | 29^th^ August 1999 |

*Table S3: Discarded and retained sample points designated ‘inside’ and ‘outside’ the target wetland areas*.

| **Site** | **Number of discarded points** | **Number of retained points** |
| --- | --- | --- |
| Okavango – inside | 11 | 189 |
| Okavango – outside | 30 | 170 |


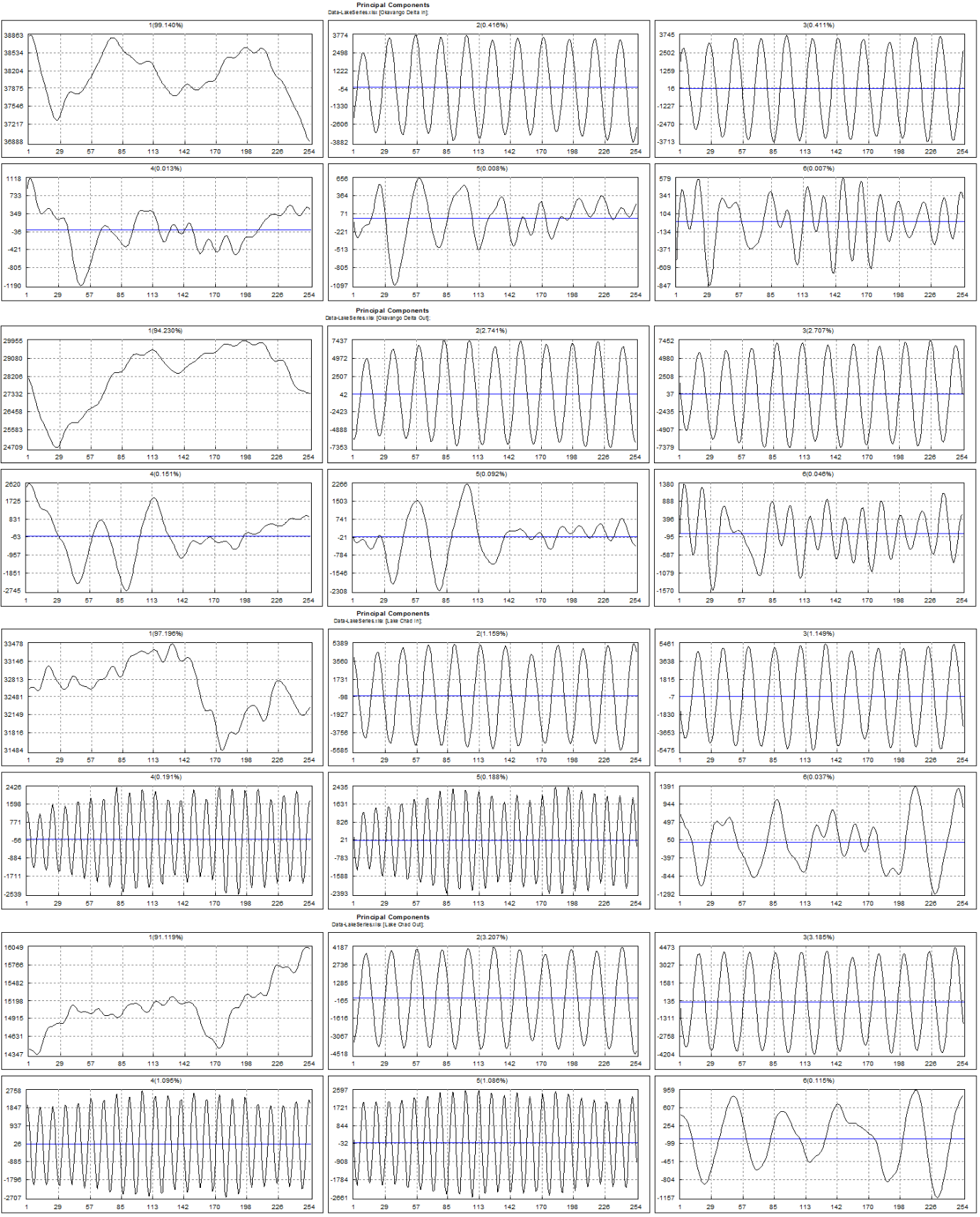


*Figure S3: Principal components related to the first 6 eigentriples for each series.*


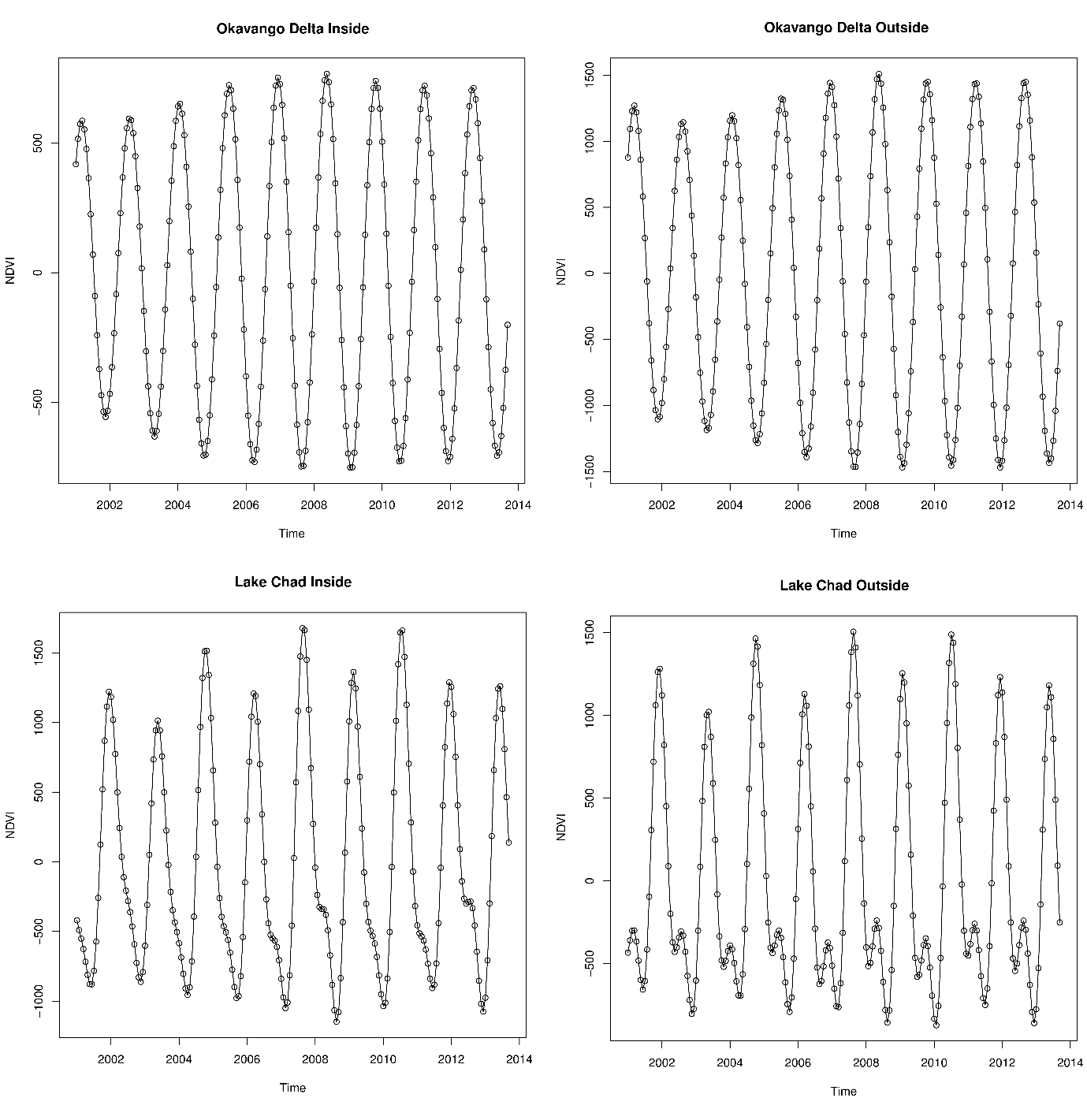


*Figure S4: Oscillation extraction (Okavango Delta: eigentriples 2–3|*


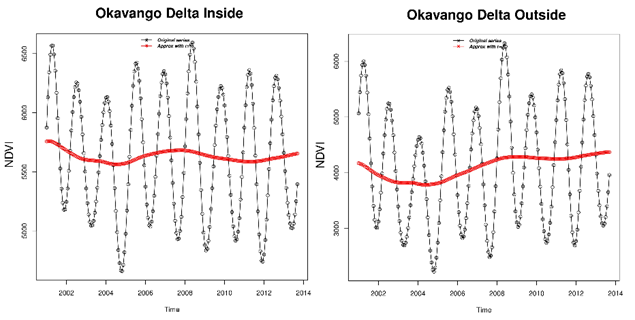


*Figure S5: Trend extraction (first eigentriple) for each series.*


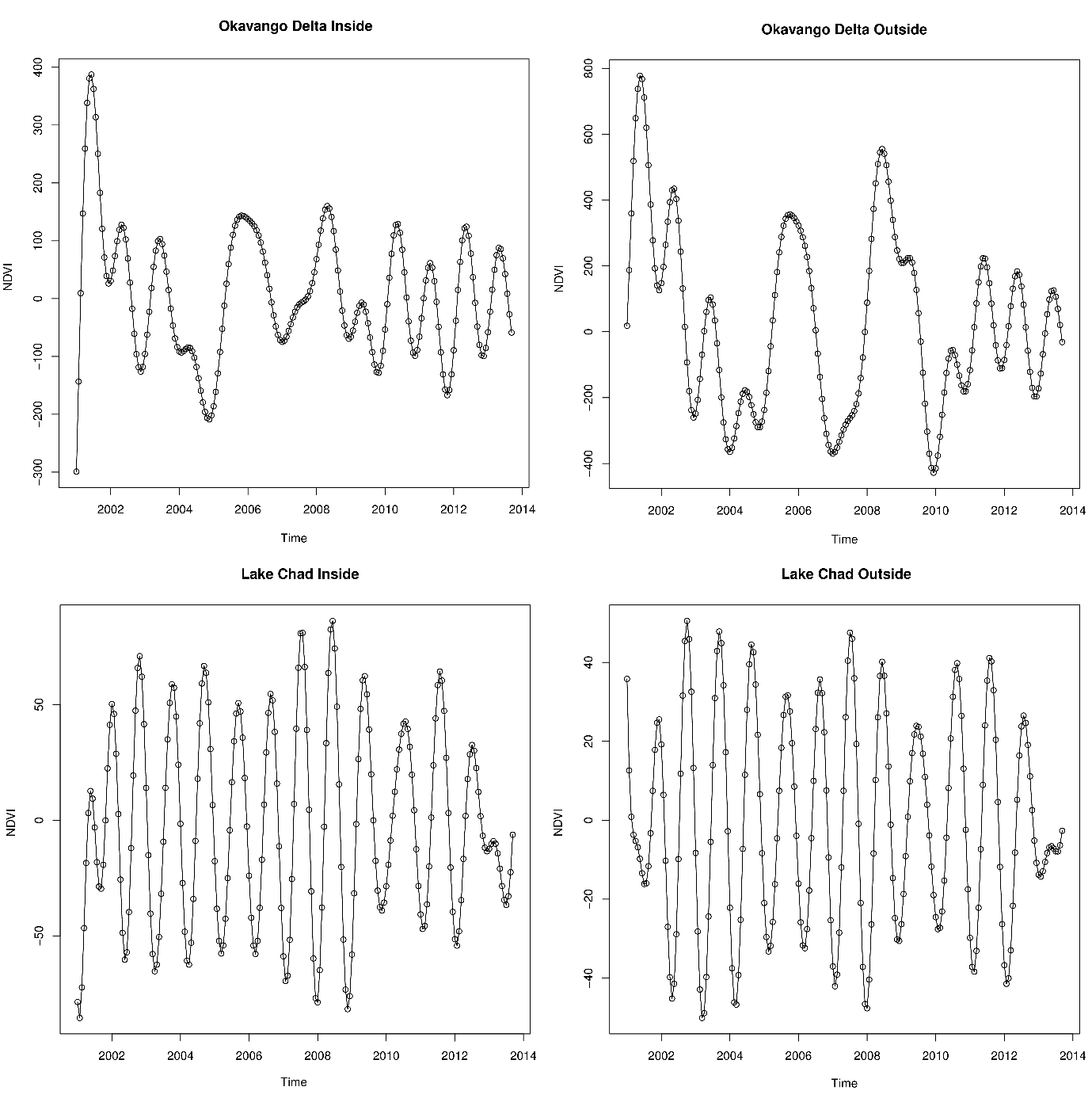


*Figure S6: Residual series (Okavango Delta: eigentriples 4–46)*

*
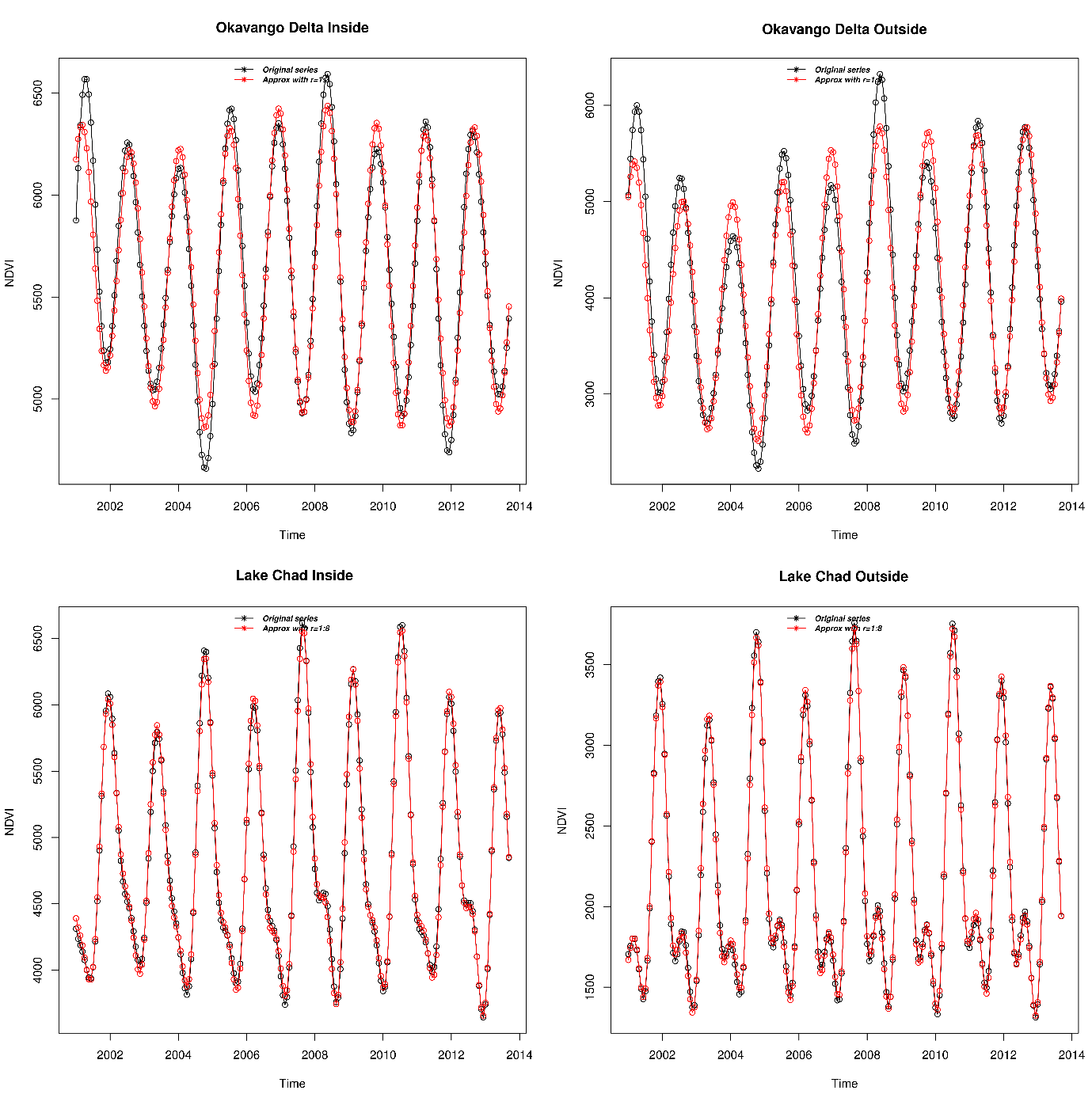
*

*Figure S7: Reconstructed series (Okavango Delta: eigentriples 1–3)*
